# Social Cognition and Neurocognition in Schizophrenia and Healthy Controls: Intercorrelations of Performance and Effects of Manipulations Aimed at Increasing Task Difficulty

**DOI:** 10.3389/fpsyt.2018.00356

**Published:** 2018-08-07

**Authors:** Elizabeth Deckler, Gabrielle E. Hodgins, Amy E. Pinkham, David L. Penn, Philip D. Harvey

**Affiliations:** ^1^Department of Psychiatry and Behavioral Sciences, University of Miami Miller School of Medicine, Miami, FL, United States; ^2^School of Behavioral and Brain Sciences, The University of Texas at Dallas, Richardson, TX, United States; ^3^Department of Psychiatry, University of Texas Southwestern Medical School, Dallas, TX, United States; ^4^Department of Psychology, University of North Carolina, Chapel Hill, NC, United States; ^5^Department of Psychology, Australian Catholic University, Melbourne, VIC, Australia; ^6^Research Service, Miami VA Healthcare System, Miami, FL, United States

**Keywords:** multivariate analysis, social cognition, neurocognition, disability, task difficulty

## Abstract

Social cognition (SC) and neurocognition appear to predict different aspects of functional outcome in people with schizophrenia. However, the correlations between performance on these domains have not been tested extensively and compared cross-diagnostically with healthy controls. Further, some social cognitive measures appeared to have potential ceiling effects, particularly for healthy people, in previous research, so increasing their difficulty is of interest. In this paper we report on two studies wherein we examined the correlations between neurocognitive ability and performance on SC tests. In the first study the correlations between measures of social perception, emotion processing, and theory of mind and performance on a brief neuropsychological (NP) assessment were examined in 179 schizophrenia (SCZ) patients and 104 healthy controls (HC). In the second study, we instructed participants to perform a subset of the tasks as rapidly as possible in order to increase task difficulty, and we examined the effects of those instructions on task difficulty, task psychometrics, and correlations between SC and NP tests in 218 SCZ patients and 154 HC. In the first study, both HC and SCZ manifested a domain specific pattern of correlation between NP and SC test performance. Controlling for group differences in NP performance did not eliminate SC performance differences between the groups. In the second study, no differences in task performance, intercorrelations other SC tests, or test-retest stability were induced by the difficulty manipulation in the samples who performed the tasks with speed demands compared to the performance of the previous sample. These data suggest that simple manipulations aimed at increasing task difficulty may not have the desired effect and that despite consistent correlations between SC and NP test performance, impairments in social cognitive functioning are not fully explained by NP performance deficits.

## Introduction

The relationship between neurocognition, social cognition and everyday functional outcome within the population of people living with schizophrenia and other severe mental illnesses has recently received considerable attention (1, 2). Social cognition, an array of cognitively demanding, socially relevant domains, includes such processes as emotion perception, awareness of others' thoughts and intentions, and social problem solving (3). There is an established correlation between performance on social cognitive tests and some elements of social outcome in people with schizophrenia. Furthermore, there is research which shows that patients can benefit from specific training geared toward enhancing social cognition (4, 5). Previous research has also suggested that negative symptoms have a direct adverse effect on the real-world functioning of these patients (6, 7), which may be combined with the influences of social cognition on social outcomes (8).

Given that neurocognitive deficits are central symptoms of schizophrenia, the correlation between neurocognitive abilities and outcomes in people with schizophrenia has been regularly investigated. There is some lack of clarity, however, regarding the relationship between social cognition, neurocognition, and everyday functioning in people with schizophrenia. One of the main topics of current interest is whether social cognition and neurocognition are independent factors, or domains, with differential effects on different domains of functioning. Earlier research has shown that the two have divergent correlations with social outcomes. For instance, a large-scale meta-analysis showed that social cognitive performance was more consistently associated with social functioning outcomes than neurocognition, across a wide range of measures of both constructs (1). Other research has suggested that there is a much smaller correlation between neurocognition, as well as associated functional capacity measures, and real world social outcomes compared to the correlation between neurocognition and functional capacity and vocational functioning and everyday activities (6).

In contrast, performance-based measures of social cognition and skills competence combine to predict a very reasonable amount of variance in social outcomes among people with schizophrenia (9). However, in this last study, the correlation between neurocognition and social cognition was substantial, sharing as much as 30% of the variance or more. Thus, divergence in prediction of functional outcomes occurs in the context of substantial correlations between social cognition and neurocognition. In considering the possible functional relationships of these domains, Lindenmeyer et al. (10) reported that enhancement of neurocognition in patients with schizophrenia using computerized cognitive training (CCT) accelerated the rate of acquisition of social cognitive skills. A follow-up study (11) suggested that combined social cognitive and neurocognitive training led to greater improvement in neurocognitive functioning than neurocognitively focused training alone. Thus, at the very least, having greater neurocognitive ability leads to better outcomes while learning social cognitive skills and it is possible that there are substantial overlaps between these domains.

It is widely understood that neurocognitive tasks are multifactorial and that completing complex cognitive tasks involves competency in multiple neurocognitive abilities. These other abilities can include memory functions, processing speed, and reasoning and problem-solving abilities. Prior research shows that people with schizophrenia fail to employ the executive functioning strategies used by healthy people when solving neurocognitive problems (12). Specifically, healthy people were found to use executive functioning strategies to approach performing a symbol coding task, while schizophrenia patients did not appear to approach the task strategically. Not only did these patients fail to use an executive functioning strategy, they also failed to use any sort of systematic processing strategy. This is interesting because symbol coding is known to be the most impaired cognitive domain among people with schizophrenia (13). It is also the most functionally relevant cognitive domain (14).

In our multisite and multiphase Social Cognition Psychometric Evaluation (SCOPE) study, participants, including healthy controls and people with schizophrenia, were given a battery of tests that evaluated neurocognition and social cognition, allowing us to examine intercorrelations of social cognitive and neuropsychological performance. Various aspects of social cognitive performance appear, at least superficially, to possibly require different neurocognitive abilities for efficient performance. These different requirements have clear ecological validity for real world functioning. Affect displays are fleeting (likely requiring competence in processing speed); socially focused verbal interactions involve having knowledge of previous statements (likely requiring competence in episodic memory); and a recollection of immediately prior information (which is a verbal working memory function) would facilitate performance on many different social cognitive tests. Thus, it would appear that performance on certain social cognitive tests requires better processing speed. Other tests might require utilizing verbal working memory or episodic memory strategies for optimal performance.

This study uses state of the art social cognitive assessments and relates them to performance on neurocognitive tests, comparing healthy controls and people with schizophrenia. The reason that neurocognitive deficits could be suspected to underlie social cognitive deficits is that neurocognitive impairments appear to be much more substantial in magnitude than social cognitive deficits. For instance, in the first SCOPE study (15), the most impaired social cognitive domain had an effect size for comparison with the performance of HC of *d* = −1.05, with mean effect size across 10 measures of *d* = −0.69. In a study of 2,616 patients with schizophrenia (16), the least impaired neurocognitive domain had an effect size of *d* = −0.99, the mean of 10 different neurocognitive tests was *d* = −1.7, and a weighted composite had an effect size of *d* = −2.2. Thus, substantially impaired neurocognitive performance might lead to challenges in performing other cognitively demanding tests, even if their demands appear to be primarily social in nature.

Previously we had reported on the joint influence of negative symptoms, social cognition and neurocognition (8) on social outcomes in schizophrenia. As our interest in this paper is comparison of healthy controls and patients in terms of social cognition and neurocognition, and because healthy controls were not rated for negative symptoms, we do not address the influences of negative symptoms here.

Here, we present analyses addressing these questions using data from two different studies whose primary outcomes were previously published. In the first study, we examined the importance of several different neurocognitive performance domains, including working memory, processing speed, and verbal learning, for predicting performance on a set of social cognitive tests in healthy controls and people with schizophrenia. For these analyses, we had two main hypotheses. Our first hypothesis was that participants with faster processing speed would perform better on emotional recognition tests that involve rapidly presented visual stimuli. Our second hypothesis was that social cognitive tests involving the processing of verbal information, such as the Hinting test, would require participants to have better verbal memory performance. We performed correlational analyses within the two subject samples to address these questions. We also tested the hypothesis that neurocognitive limitations contributed to group differences in social cognition by adjusting group differences in social cognition for differences in neurocognitive performance.

In the second study, healthy controls and patients were instructed to perform three of the social cognitive tests from the first study as rapidly as possible without sacrificing accuracy. As noted above (15) certain social cognitive tests had relatively high levels of performance on the part of schizophrenia patients in the first SCOPE study, particularly in reference to differences between patients and controls in neurocognitive measures. These high levels of performance have the potential to suppress group differences and also lead to reduced variance in social cognitive performance. This reduced variance may also suppress the predictive utility of these tasks in terms of everyday outcomes. Thus, the goal of this manipulation was to make the tasks more challenging for all participants (17). We examined the correlations between neurocognitive performance and these three tasks in both subject populations. We also examined the general influence of the speeded performance requirements by comparing task performance across the two studies with and without speed instructions. It was our hypothesis that people with schizophrenia would be less able to respond to speed demands than healthy individuals and that their performance would be more adversely impacted as a result. We also expected that the speed performance demands might have an impact on the psychometric characteristics of the tests as well.

## Study 1

### Methods

#### Participants

These data come from Phase I of the Social Cognition Psychometric Evaluation (SCOPE) study (15), a multisite study, performed at the University of Miami Miller School of Medicine (UM) and Southern Methodist University (SMU). At UM, patients were recruited from both the Miami Veterans' Hospital and Jackson Memorial Hospital-University of Miami Medical Center. At SMU, patients were recruited from Metrocare Services, a non-profit mental health services provider organization located in Dallas County, Texas. Community advertisements were used to recruit healthy controls at both sites.

Patients were required to have a DSM-IV diagnosis of schizophrenia or schizoaffective disorder, with this diagnosis confirmed by clinical interview utilizing the SCID Psychosis Module (18) and the MINI International Neuropsychiatric Inventory (19). In addition to a DSM-IV diagnosis of schizophrenia or schizoaffective disorder, patients had to be on a regular medication schedule for at least 6 weeks with no dose changes for at least 2 weeks. Patients also could not have been hospitalized in the past 2 months.

To ensure that the healthy controls did not have a history of significant psychopathology, they were also interviewed for the presence of major Axis I or II disorders. Exclusion criteria for both groups included: (1) presence or history of pervasive developmental disorder or mental retardation (defined as IQ < 70) by DSM-IV criteria, (2) presence or history of medical or neurological disorders that may affect brain function (e.g., seizures, CNS tumors, or loss of consciousness for 15 min or more), (3) presence of sensory limitation including visual (e.g., blindness, glaucoma, vision uncorrectable to 20/40) or hearing impairments that interfere with assessment, (4) no proficiency in English, (5) presence of substance abuse in the past month, and (6) presence of substance dependence not in remission for the past 6 months.

In this study, there were 179 patients with schizophrenia/schizoaffective disorder and 104 healthy controls. As the information on their demographic characteristics was published previously, it is presented in Supplemental Table 1. All participants provided signed informed consent, and this study was reviewed by the IRBs at all research sites.

### Measures

#### Social cognition measures

##### Emotion processing

*Bell lysaker emotion recognition task [BLERT; [**20**]]*. The BLERT measures the ability to correctly identify seven emotional states: happiness, sadness, fear, disgust, surprise, anger, or no emotion. Stimuli are presented on a monitor and consist of videos depicting these different emotions. The dependent variable was the total correct out of 21 possible items.

*Penn emotion recognition text [ER-40; [**21**]]*. The ER-40 measures the ability to accurately identify both high-intensity and low intensity emotions conveyed in static photographs of faces presented on a computer monitor in a PowerPoint format. Facial expressions include happiness, sadness, anger, fear, and no emotion. The dependent variable is the total correct out of a possible score of 40.

##### Social perception

*Relationships across domains, abbreviated version [RAD; [**22**]]*. The RAD measures competence in the perception of four relational models: communal sharing, authority ranking, equality matching, and market pricing using 15 written vignettes involving different male-female dyads that represent one of the relational models. The dependent variable for this test is the total number correct out of 45.

##### Theory of mind/mental state attribution

*Reading the mind in the eyes test [Eyes; [**23**]]*. The Eyes Test measures the participant's capacity to determine the mental state of others from expressions in the eye region of the face. This is done by viewing 36 photos of the eye region of different faces. The dependent variable is the total number correct.

*The awareness of social inferences test, part III [TASIT; [**24**]]*. The TASIT assesses detection of lies and sarcasm using 16 videos of various social interactions. After viewing each video, participants respond to four questions about the intentions of the characters in a yes/no format for a total of 64 possible correct responses.

*Hinting task [**25**]*. The Hinting Task examines the ability of individuals to infer the true intent of indirect speech by using 10 short verbal passages that present an interaction between two characters. Each passage ends with one of the characters dropping a hint, and participants must state what the character wanted. The dependent variable is the total score, out of a possible score of 20.

##### Neurocognitive measures

Participants completed a subset of the MATRICS Consensus Cognitive Battery (26) including Trail Making Test-Part A, BACS-Symbol Coding, Category Fluency-Animal Naming, Letter-Number Span, and the Hopkins Verbal Learning Test-Revised. We also created a composite measure by averaging the t-scores generated by the MCCB scoring program for the tests that we administered.

Table 1 presents the scores on the neurocognitive and social cognitive tests in this study for the two subject samples.

**Table 1:** Descriptive statistics of neurocognitive and social cognitive tasks for patients and healthy controls.

	**Patients**		**Controls**	
**Task**	***N*** = **179**		***N*** = **104**	
	**Mean**	**SD**	**Mean**	**SD**
TRAILS A: time in seconds	41.06	18.78	30.72	12.10
Symbol coding	42.18	11.78	53.99	14.00
Hopkins verbal learning test total learning	20.27	5.37	24.85	4.45
Letter number sequencing	11.37	4.07	13.85	3.85
Animal fluency	18.44	5.12	21.98	6.36
Bell-Lysaker emotion recognition test	13.17	3.88	15.75	2.88
ER-40 total number correct	29.55	5.40	32.80	3.54
Eyes test	20.14	5.46	23.55	4.62
Hinting test	13.59	3.87	16.82	2.05
RAD total score	24.79	5.79	29.87	5.21
TASIT total score	44.43	7.64	51.48	5.62

*SD, standard deviation*.

#### Procedures

The participants in the study were evaluated twice because one of the goals of the larger study was to examined test-retest reliability. The first evaluation was a baseline assessment. The second evaluation was a retest assessment performed between 2 and 4 weeks after the baseline assessment (mean = 17 days). Data from the first assessment are used in this study. During the baseline assessment, all participants provided informed consent and completed neurocognitive, social cognitive, and functional outcome evaluations. For participants with schizophrenia or schizoaffective disorder, a diagnostic assessment, and evaluation of symptom severity were performed during the baseline assessment. Symptom severity was measured using the Positive and Negative Syndrome Scale. Diagnostic and symptom raters were trained using the established procedures at each site to guarantee reliability.

#### Statistical analyses

Our primary interest was the correlation between neurocognitive and social cognitive performance in the two subject groups. We used Pearson correlation analyses to examine the correlations between the two sets of variables. Correlations were calculated separately in the two samples and tested for the significance of the differences using Fisher's *r* to *z* transformation. Following those analyses, we performed a canonical analysis in order to examine the global patterns of intercorrelation between neurocognition and social cognition in the two samples. We then used regression analyses to examine the independent prediction of the social cognitive variables by the neurocognitive variables. We were also interested in the extent to which neurocognitive performance was associated with the previously reported differences in social cognitive performance between the groups. In order to test this question, we used a multivariate analysis of variance (MANOVA) with and without composite cognitive performance as a covariate.

### Results

Table 2 presents the correlations between neurocognitive performance and social cognitive performance in the two subject samples. As can be seen in the table, neurocognitive performance was consistently correlated with social cognitive performance in the healthy sample. In fact, only 6 of 36 correlations were not significant. For the schizophrenia patients, essentially the same relationships were found. All of the neurocognitive variables were correlated with at least some of the social cognitive variables and the composite score shared between 9 and 17% of the variance with the social cognitive variables, which was actually greater than that seen with the healthy controls. With Fisher's *r* to *Z* transformation there were four correlations where the differences were significant. In all cases, the correlations were larger in patients than in healthy controls.

**Table 2:** Correlations between neurocognitive test performance and performance on social cognitive measures.

	**ER-40**	**BLERT**	**EYES**	**HINTING**	**RAD**	**TASIT**
**TRAIL MAKING PART A**						
Healthy control	−0.24^*^	−0.36^**^	−0.30^**^	0.003	−0.29^**^	−0.40^**^
Schizophrenia patient	−0.18^*^	−0.24^**^	−0.27^**^	−0.13	−0.19^*^	−0.28^*^
**BACS SYMBOL CODING**						
Healthy control	0.25^*^	0.38^**^	0.40^**^	0.03^a^	0.34^**^	0.36^**^
Schizophrenia patient	0.36^**^	0.36^**^	0.44^**^	0.24^**^	0.38^**^	0.42^**^
**ANIMAL NAMING**						
Healthy control	0.10	0.23^**^	0.23^*^	0.25^*^	0.22^*^	0.24^*^
Schizophrenia patient	0.18^*^	0.15	0.26^**^	0.21^*^	0.28^**^	0.24^**^
**HVLT TOTAL LEARNING**						
Healthy control	0.21^*^	0.35^**^	0.35^**^	0.22^*^	0.35**^a^	0.37^**^
Schizophrenia patient	0.38^**^	0.35^**^	0.35^**^	0.35^**^	0.52^**^	0.47^**^
**MARYLAND LNS TASK**						
Healthy control	0.08^a^	0.36^**^	0.39^**^	0.09^a^	0.30**^a^	0.39^**^
Schizophrenia patient	0.44^**^	0.44^**^	0.54^**^	0.39^**^	0.57^**^	0.47^**^
**COGNITIVE COMPOSITE**						
Healthy control	0.13	0.27^*^	0.34^**^	0.21^*^	0.27^*^	0.23^*^
Schizophrenia patient	0.33^**^	0.35^**^	0.44^**^	0.30^*^	0.37^**^	0.44^*^

* *p < 0.05*;

** *p < 0.01*;

a *Correlations significantly different at p < 0.05*.

Supplemental Table 2 presents the intercorrelations between the social cognitive variables. As can be seen in the table, the measures were moderately intercorrelated in the schizophrenia sample, with 14/15 correlations significant. For the healthy controls, 10/15 correlations were significant and magnitude of the correlations was consistently smaller than that seen in the patients. In particularly the hinting task was correlated only with the RAD in healthy controls.

For our canonical analysis, we placed all six social cognition variables on one side of the equation and all four neurocognition variables on the other. The best fitting solution for healthy controls was a single canonical root, *F*_(30, 366)_ = 2.04, *p* = 0.001. A second possible root was not close to statistical significance, *F*_(20, 306)_ = 0.89, *p* = 0.60. The variance accounted for was 0.42 for the social cognition set, 0.52 for the neurocognition set and, most importantly, the cross correlations of set 1 × set 2 accounted for 33% of the variance. For the schizophrenia patients the best fitting solution was also a single root, *F*_(30, 464)_ = 5.11, *p* < 0.001, with second root not close to significance, *F*_(20, 538)_ = 1.10, *p* = 0.35. Similarly, set 1 accounted for 0.55 of the variance, set 2 accounted for 0.44 and the cross correlations accounted for 0.50 in the variance. For both samples, the results suggest that there is a similar pattern of strong correlation between the sets of variables, consistent with homogenous relationship between neurocognition and social cognition.

We computed regression analyses predicting each of the social cognitive variables with the neurocognitive variables in the healthy control and patient samples. The analyses for each social cognition variable incorporated all of the NP variables into a stepwise regression model. The results of these stepwise analyses are presented in Table 3. The social cognition tests other than Hinting and the RAD were significantly predicted in both groups by various NP tests. In fact, the amount of shared variance was in some cases substantial. For all variables, the shared variance between cognitive tests and social cognitive tests was greater in the schizophrenia sample. Tests with intrinsic speed components, including the BLERT, ER-40, and the TASIT, correlated with processing speed measures. Tests with verbal demands, such as the hinting test and RAD, correlated with animal naming or HVLT scores. Verbal working memory performance contributed independent variance to 6/6 tasks in the schizophrenia patients and to the BLERT, Eyes, and TASIT in the healthy controls as well.

**Table 3:** Regression analyses predicting social cognitive performance with neurocognitive tests.

		**Healthy controls**					**Schizophrenia patients**			
**Social cognitive variable**		***t***	***p***	***R***${}_{\text{Change}}^{2}$	***R***^2^_Total_		***t***	***p***	***R***${}_{\text{Change}}^{2}$	***R***^2^_Total_
	**NP variable**					**NP variable**				
**BLERT**										
Step 1	Symbol coding	4.08	0.001	0.14	0.14	Letter-number span	4.90	0.001	0.20	0.20
Step 2	Letter-number span	2.67	0.009	0.05	0.19	Symbol coding	2.45	0.02	0.02	0.22
**ER-40**										
Step 1	Symbol coding	2.61	0.01	0.06	0.06	Letter-number span	5.11	0.001	0.21	0.21
Step 2	–					Symbol coding	2.57	0.01	0.03	0.24
**EYES TEST**										
Step 1	Symbol coding	2.83	0.006	0.16	0.16	Letter-number span	6.40	0.001	0.29	0.29
Step 2	Letter-number span	2.71	0.008	0.06	0.22	Symbol coding	3.50	0.001	0.05	0.34
**HINTING TEST**										
Step 1	Animal naming	2.54	0.01	0.06	0.06	Letter-number span	3.41	0.001	0.15	0.15
Step 2						HVLT	2.14	0.04	0.02	0.18
**TASIT**										
Step 1	Trail making part A	−4.40	0.001	0.16	0.16	Letter-number span	3.48	0.001	0.22	0.22
Step 2	Letter-number span	3.09	0.003	0.07	0.23	Symbol coding	3.18	0.002	0.07	0.32
**RAD**										
Step 1	HVLT	3.76	0.001	0.12	0.12	Letter-number span	5.62	0.001	0.33	0.33
						HVLT	3.96	0.001	0.06	0.39

Given that neurocognitive variables in schizophrenia are often unifactorial, we computed collinearity statistics for the neurocognitive predictors with the SPSS (V24) collinearity diagnostics routine. The critical statistics are “condition indices” which are computed as the square roots of the ratios of the largest eigenvalue to each successive eigenvalue. Values >15 indicate a possible problem with collinearity and >30 reflects a serious problem. For the schizophrenia patients, across the six regression analyses, there were no identified dimensions that exceeded the threshold of 15. In the HC sample, there were two of the six dimensions over the threshold of 15, but the highest value was 25. Running an outlier test with a cutoff of 3.0 SD led to the detection of two SCZ patients and no HC. As patients with schizophrenia can perform 3.0 SD below normative standards on individual tests (16), this finding does not reflect a major concern.

Given the substantial variance shared between neurocognitive tests and the social cognitive and our previous reports that the HC outperformed the SCZ patients on all tasks in both domains, we computed our planned Multivariate Analysis of Variance (MANOVA). In this, we entered all six social cognition variables as dependent variables and used diagnostic group as the between-subjects factor. We also used the composite measure of neurocognitive performance described above as the cognitive performance covariate.

The overall effect of diagnosis on the social cognitive measures was significant, Wilks Lambda = 0.881, *F*_(6, 269)_ = 6.08, *p* < 0.001. The effect of the composite cognitive covariate was also significant, Wilks Lambda = 0.881, *F*_(6, 269)_ = 15.23, *p* < 0.001. Other than the eyes test, *F*_(1, 269)_ = 2.15, *p* = 0.16, all of the other social cognitive variables still differed between the HC and SCZ samples with the effects of global cognitive performance controlled (all *F* > 5.01, all *P* < 0.025). If the analysis had been run without cognitive performance as a covariate, the group differences on all of the social cognitive measures would have been substantially greater: all *F* > 28.73, all *p* < 0.001.

### Discussion: study 1

Healthy controls and patients with schizophrenia manifested quite similar patterns of correlation between neurocognitive and social cognitive test performance. There was a large and consistent correlation between these two performance domains in schizophrenia, similar to that seen in the healthy controls. The correlations on the part of both groups, but particularly the schizophrenia patients, suggested substantial contributions of processing speed and verbal working memory. The social cognitive tests requiring verbal processing, specifically the hinting test and the RAD, were associated with verbally relevant abilities, including animal naming in the HC and HVLT and verbal working memory performance, across the two participant groups. These findings are quite consistent with earlier studies showing that the greatest performance deficits in schizophrenia patients are in domains of processing speed, working memory, and verbal memory. Better performance on tests of these abilities was associated with better social cognitive performance.

Adjusting for neurocognitive performance did not eliminate differences between the groups other than on the eyes test. This test had the smallest effect size between groups of all of the social cognitive measures in the parent study (15). It also shared 34% of the variance with cognitive performance in the patient sample in that study, suggesting that future research on this test may need to consider its relationship with neuropsychological performance. However, adjusting for neurocognitive test performance notably reduced group differences in social cognitive test performance across the measures as a group. This overlap needs to be carefully considered in future studies and the contributions of both neurocognitive and social cognitive should be considered when attempting to remediate impairments in everyday functioning. The findings of the canonical analyses are consistent with this interpretation, wherein both groups had a solution that suggested joint loading of social cognition and neurocognition on a single canonical latent factor.

## Study 2

### Background

In a second study, we attempted to increase the difficulty of the social cognitive assessments in order to improve task sensitivity to group differences. We asked participants to perform three of the tests, the BLERT, the ER-40, and the TASIT, as rapidly as possible without sacrificing accuracy. We were primarily interested in whether these instructions led to changes in overall performance and in which of the subject groups, in that we hoped for an increase in difficulty for healthy people and schizophrenia patients. We were also interested in finding out if this manipulation would lead to changes in other task characteristics such as test-retest reliability, intercorrelations between the social cognitive variables, and correlations between the neurocognitive and social cognitive measures. We took the data from the first study, where tasks were administered with standard instructions, and compared them to the results of the tasks administered in the second study with speeded performance instructions. To make it clear, the participants in the first study were all tested twice with standard instructions and the subjects in the second study were all tested twice with speeded instructions. We were also interested in understanding if this manipulation would affect the likelihood that the participants, either healthy controls or schizophrenia patients, would employ different neurocognitive strategies to solve the social cognitive tests.

### Methods

#### Participants

This study included healthy controls and patients with schizophrenia/schizoaffective disorder. The results of their performance on social cognitive and neurocognitive tests were already published (27). The regarding demographic characteristics has also been published, and it is presented in Supplemental Table 3 of this paper.

Data collection for this study occurred at three sites: The University of Texas at Dallas (UTD), The University of Miami Miller School of Medicine (UM), and The University of North Carolina at Chapel Hill (UNC). Participants were stable outpatients with diagnoses of schizophrenia or schizoaffective disorder (*n* = 218) and healthy controls (*n* = 154). UNC patients were recruited from the Schizophrenia Treatment and Evaluation Program (STEP) in Carrboro, NC and the Clinical Research Unit (CRU) in Raleigh, NC. At all sites, healthy controls were recruited via community advertisements. All participants provided signed informed consent, and this study was reviewed by the IRBs at all research sites.

#### Assessments

Participants completed modified versions of the BLERT, the ER-40, and the TASIT. As these were the only tasks whose instructions changed, we examined these tasks only. The three tasks were modified in two ways from the standard administration. First, participants were instructed to respond as rapidly as possible without sacrificing accuracy. Second, after making their response, participants rated how confident they were that their response was correct on a scale from 0 (not at all confident) to 100 (extremely confident). Response time to answer each item was recorded from stimulus onset to when the participant provided their answer. Neurocognitive performance was assessed with the same measures as the previous study.

#### Procedures

As noted above, participants were asked to perform the tests as rapidly and efficiently as possible. In this study, patients and controls were examined two times with the new instructions used at both times. The two assessments were performed an average of 17 days apart. The data from study 1 above were used to reference performance with standard instructions.

#### Analyses

We examined the performance across the two studies on the three tests that were administered with speed instructions in the second study. None of the research participants participated in both studies, so this is not a repeated-measures design. In order to examine the performance on the tests, we performed a group (HC, SCZ) x instruction condition (Standard, Speeded) multivariate analysis of variance (MANOVA) for total performance for the three social cognitive tests across the two studies. We then performed a correlational analysis using Pearson Correlations, examining the correlations between the neuropsychological measures and social cognitive measures in the second study. We also examined the intercorrelations of the social cognitive measures and their test-retest reliability in both studies.

### Results

Scores on the social cognitive tests across the two studies are presented in Table 4. The results of the MANOVA found a statistically significant effect of group, *F*_(3, 648)_ = 48.06, *p* < 0.001. There was, however, no significant effect of instructional condition, *F*_(3, 648)_ = 1.87, *p* = 0.13, or interaction of instructional condition x group, *F*_(3, 648)_ = 1.04, *p* = 0.37, suggesting that there were no performance differences associated with instructional conditions in either of the samples and across all of the tests.

**Table 4:** Performance on social cognition tests with and without speed demands.

	**Standard administration**				**With speed demands**			
	**HC (*****n*** = **104)**		**SC (*****n*** = **179)**		**HC (*****n*** = **154)**		**SC (*****n*** = **218)**	
	**Mean**	***SD***	**Mean**	***SD***	**Mean**	***SD***	**Mean**	***SD***
BLERT	15.75	2.88	13.17	3.877	15.91	2.70	13.93	4.02
ER-40	32.80	3.537	29.55	5.404	32.94	3.19	31.12	4.28
TASIT	51.48	5.62	44.43	7.64	51.37	6.71	44.99	7.45

Table 5 presents the results of the correlational analyses. As can be seen in the table, there were no notable differences in the test-retest reliability estimates across conditions/studies. Further, the intercorrelations between the social cognitive measures were highly similar across the two instructional conditions. The correlations presented in Table 6 between neuropsychological test performance and social cognition performance were consistent across the two studies for the healthy controls, with 13/15 correlations between social cognitive and neurocognitive performance significant at *p* < 0.05 in both studies. For the patients, there were 14/15 correlations between social cognition and neurocognition in study 1 found to be significant at *p* < 0.05 and 15/15 that were significant at *p* < 0.01 in study 2. Tests using the *r* to *z* transformation found that there were no statistically significant differences between any of the correlation coefficients across the two studies, in either of the participant groups.

**Table 5:** Psychometric properties of the tasks across instructional demands test retest stability of performance and intercorrelations of tests.

	**Standard administration**				**With speed demands**			
	**HC**		**SC**		**HC**		**SC**	
	***r***	***p***	***r***	***p***	***r***	***p***	***r***	***p***
BLERT	0.68	0.001	0.70	0.001	0.62	0.001	0.81	0.001
ER-40	0.75	0.001	0.75	0.001	0.68	0.001	0.71	0.001
TASIT	0.54	0.001	0.60	0.001	0.53	0.001	0.64	0.001
	**HC (*****n*** = **104)**		**SC** ***N*** = **179**		**HC** ***N*** = **154**		**SC** ***n*** = **218**	
	**Standard administration**			**With speed demands**		
	**BLERT**	**ER-40**		**TASIT**	**BLERT**	**ER-40**		**TASIT**
BLERT	1.00		0.35^**^	0.50^**^	1.00	0.34^**^		0.38^**^
ER-40	0.59^**^		1.00	0.22^*^	0.65^**^	1.00		0.38^**^
TASIT	0.52^**^		0.51^**^	1.00	0.53^**^	0.50^**^		1.00

* *Pearson product moment correlation is significant at the 0.05 level (2-tailed)*.

** *Correlation is significant at the 0.01 level (2-tailed)*.

*HC correlations above, SCZ patients correlations are below. No correlations differ across speed demands at p < 0.05*.

**Table 6:** Convergence of NP test performance and social cognitive performance.

	**Standard administration**						**With speed demands**					
	**HC (*****n*** = **104)**			**SCZ (*****n*** = **179)**			**HC (*****n*** = **154)**			**SCZ (*****n*** = **218)**		
	**BLERT**	**ER-40**	**TASIT**	**BLERT**	**ER-40**	**TASIT**	**BLERT**	**ER-40**	**TASIT**	**BLERT**	**ER-40**	**TASIT**
Trails	−0.36^**^	−0.24^*^	−0.40^**^	−0.24^**^	−0.18^*^	−0.28^**^	−0.32^**^	−0.14	−0.43^**^	−0.31^**^	−0.26^**^	−0.30^**^
Sym C	0.38^**^	0.25^*^	0.36^**^	0.36^**^	0.36^**^	0.42^**^	0.35^**^	0.21^**^	0.42^**^	0.44^**^	0.33^**^	0.37^**^
HVLT	0.36^**^	0.22^*^	0.37^**^	0.35^**^	0.38^**^	0.47^**^	0.180^*^	0.15	0.38^**^	0.46^**^	0.30^**^	0.46^**^
LNS	0.36^**^	0.09	0.39^**^	0.44^**^	0.44^**^	0.47^**^	0.190^*^	0.18^*^	0.50^**^	0.50^**^	0.39^**^	0.43^**^
AF	0.23^*^	0.10	0.24^*^	0.15	0.18^*^	0.24^**^	0.26^**^	0.18^*^	0.34^**^	0.44^**^	0.26^**^	0.31^**^

*Trails, trail making test part A; Sym C, symbol coding; HVLT, Hopkins verbal learning test, revised; LNS, letter number sequencing; AF, animal fluency*.

* *Correlation is significant at the 0.05 level (2-tailed)*.

** *Correlation is significant at the 0.01 level (2-tailed)*.

*No correlations differ at p < 0.05 across speed demands*.

## Discussion

In the first study people with schizophrenia manifested patterns of social cognitive performance that were correlated with neurocognitive performance in a very similar profile and magnitude compared to healthy controls. Further, adjusting for neurocognitive performance notably attenuated, but did not eliminate, the differences between healthy controls and schizophrenia patients on social cognitive tests other than on the eyes test. Thus, when considering the magnitude of social cognitive deficits in schizophrenia, the contribution of impairments in performance on neurocognitive measures must be considered.

In the second study, two important findings emerged. First is that adding speed demands to the current set of social cognitive tests had no impact on any element of performance across the tasks, including difficulty, variability, reliability, and convergent validity among social cognitive tests for either group of participants. Second is that even though processing speed deficits are the most salient impairment in schizophrenia, and the most consistent correlation of social cognitive performance in these two studies, increasing speed of processing demands did not lead to an increase in task difficulty on any tasks and did not change their psychometric characteristics for either patient group.

The reasons for the failure of this manipulation are not clear. It is possible that these tests are easy enough for healthy controls so that doing them faster does not change the difficulty level. It is also possible that the HC in study 1 were spontaneously performing the tasks as rapidly as they could. For patients, it is known that instructional manipulations do not necessarily lead to performance changes, for better or worse. In memory research, telling people with schizophrenia to encode information does not improve performance, whereas incidental encoding manipulations lead to performance improvements (28, 29). We also do not know if the people with schizophrenia were also attempting to work rapidly in study 1.

In terms of other strategies to increase the difficulty of social cognitive tests, there are some possibilities and some contraindications. Increasing the length of assessments is not a viable solution, because the tests that were rated by testers and patients as most unfeasible in the two studies (15, 27) were the longer ones. Increasing stimulus complexity is also unlikely to be viable, in that patients already required definitions of some of the terms of used in the Eyes test (27). One strategy that might be viable would be to create an *a priori* definition of items that are not adequately challenging and eliminate them from analysis. In our pilot study with the speeded instructions (17), we found that healthy controls were correct on 73% of the BLERT items and patients on 63%. With seven different emotions represented on the BLERT, this means that performance was 60% better than chance for HC and 50% better for patients. In the second study presented here, healthy controls averaged over 80% correct on 9 of the 21 BLERT items and patients were correct over 80% of the time on 5 of the 21 items and over 70% correct on 4 more.

It has been noted previously that the everyday functional correlates of social cognitive performance and neurocognitive abilities may not manifest substantial overlap in people with schizophrenia. However, the current results demonstrate that select neurocognitive abilities do share variance with social cognitive performance in ways that are predictable (e.g., verbal memory correlates with performance on social cognitive tasks with high verbal demands) and that may be informative in future treatment studies. As previously reported by Lindenmayer et al. (10, 11), training neurocognitive skills may speed the path to social cognitive gains elsewhere. This may also suggest, consistent with our earlier arguments, Harvey and Sand (30) that augmentation of social cognitive training with pharmacological strategies aimed at neurocognitive abilities may be a feasible way forward as well.

Several recent studies have suggested that disorganization symptoms may play a role in the relationship between social cognition, neurocognition, and real-world outcomes in schizophrenia (31, 32). In those two studies, patients with more severe symptoms of disorganization manifested smaller correlations between social cognition and neurocognitive test performance. When we correlated the severity scores on conceptual disorganization with the social cognition measures (in the patients alone), we found statistically significant correlations with ER-40, BLERT, and TASIT in the first study (range *r* = 0.19–0.26, all *p* < 0.01) and in the second (range *r* = 0.15–0.25, all *p* < 0.007). Thus, symptoms of disorganization are also important to consider when evaluating whether social cognitive task performance deficits are being exaggerated by other factors.

There are some limitations in these data and of these studies. The neurocognitive assessment battery was not fully comprehensive and could not address the possibility that other cognitive ability domains not measured would have predicted social cognitive performance in study 1. The same patients were not tested twice with speeded and non-speeded processing demands, so the lack of difference might be due to differences in the samples. The size of these samples may partially overcome this concern. Further, retesting across the two processing demands would have to be counterbalanced and this was not a major part of the SCOPE III study.

## Conclusions

The results of these two studies suggest that neurocognitive and social cognitive test performance is consistently related and that at least part of the apparent social cognitive limitations seen in patients with schizophrenia may be related to impairments in neurocognitive abilities. Future research will be needed to more carefully examine the treatment implications of these findings. Also, making changes in task demands does not always ensure that there will be subsequent performance changes. Later work will have to focus on making social cognition tests more challenging with other strategies.

## Ethics statement

This study was approved by the IRBs at each of the study sites. All patients and informants signed an informant consent document in accordance with the Declaration of Helsinki.

## Author contributions

PH, AP, and DP conceived of the study and conducted it. ED and GH worked on data analyses and writing of the paper. All authors approved the final draft.

### Conflict of interest statement

PH has received consulting fees or travel reimbursements from Akili, Boehringer Ingelheim, Intra-Cellular Therapies, Lundbeck Pharma, Minerva Pharma, OtsukaAmeica, Sanofi Pharma, Sunovion Pharma, Takeda Pharma, and Teva during the past year. He has a research grant from Takeda and from the Stanley Medical Research Foundation. The remaining authors declare that the research was conducted in the absence of any commercial or financial relationships that could be construed as a potential conflict of interest. The reviewer JR and handling Editor declared their shared affiliation.
