# Supplementary material for: Social Cognition and Neurocognition in Schizophrenia and Healthy Controls: Intercorrelations of Performance and Effects of Manipulations Aimed at Increasing Task Difficulty
Source: Front Psychiatry. 2018 Aug 7;9:356. doi: 10.3389/fpsyt.2018.00356 (PMC6091232; doi:10.3389/fpsyt.2018.00356)
Supplement: Supplementary file 1 [file Data_Sheet_1.docx]

**Supplemental Table 1.**

**Participant Demographic and Clinical Characteristics Study 1**


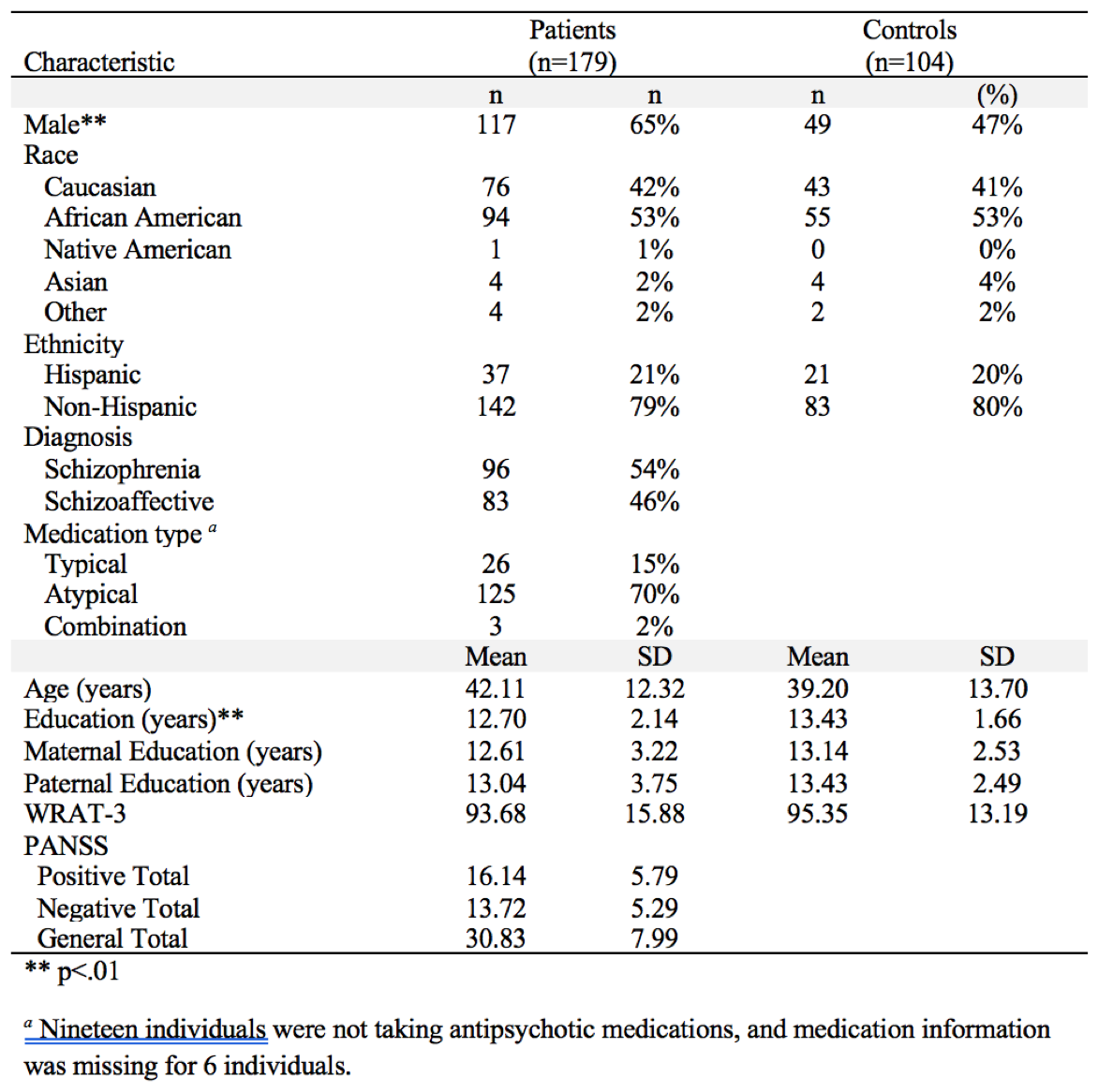
**Supplemental Table 2**

Intercorrelations of Social Cognitive Variables in Study 1.

ER-40 BLERT EYES HINTING RAD TASIT

ER-40 - - .59** .52** .31** .46** .51**

BLERT 0.35** - - .52** .37** .47** .52**

EYES 0.36** .42** - - .46** .56** .55**

HINTING -.03 .16 .14 - - .24* .18

RAD -.08 .36** .41** .24* - - .50**

TASIT 0.22* .50** .46** .18 .50** - -

Correlations For Patients Are Above the Diagonal; Correlations for HC are Below

Note. * p<.05; **p<.01

**Supplemental Table 3**

**Participant demographic and clinical characteristics Study 2**

| Characteristic | Patients  (n=218) | | Controls  (n=154) | |
| --- | --- | --- | --- | --- |
|  | n | (%) | n | (%) |
| Male | 142 | 65 | 97 | 63 |
| Race |  |  |  |  |
| Caucasian | 115 | 53 | 80 | 52 |
| African American | 87 | 40 | 62 | 40 |
| Native American | 3 | 1 | 0 | 0 |
| Asian | 6 | 3 | 4 | 3 |
| Other | 7 | 3 | 8 | 5 |
| Ethnicity |  |  |  |  |
| Hispanic | 33 | 15 | 26 | 17 |
| Non-Hispanic | 185 | 85 | 128 | 83 |
| Diagnosis |  |  |  |  |
| Schizophrenia | 112 | 51 |  |  |
| Schizoaffective  Medication type *^a^* | 106 | 49 |  |  |
| Typical | 25 | 12 |  |  |
| Atypical | 161 | 74 |  |  |
| Combination | 16 | 7 |  |  |
| No antipsychotic | 15 | 7 |  |  |
|  | Mean | SD | Mean | SD |
| Age (years) | 41.72 | 11.64 | 41.95 | 12.42 |
| Education (years)** | 13.04 | 2.49 | 14.19 | 1.91 |
| Maternal Education (years) | 13.43 | 3.61 | 13.25 | 2.93 |
| Paternal Education (years) | 13.52 | 4.19 | 13.49 | 3.26 |
| WRAT-3** | 94.78 | 14.64 | 101.11 | 11.48 |
| PANSS |  |  |  |  |
| Positive Total | 15.96 | 5.31 |  |  |
| Negative Total | 14.09 | 5.67 |  |  |
| General Total | 31.63 | 8.09 |  |  |
| CPZ equivalent | 463.64 | 422.82 |  |  |

** p<.01

*^a^* Medication information was missing for 1 patient.

Abbreviations: WRAT, Wide Range Achievement Test; PANSS, Positive and Negative Syndrome Scale; CPZ, Chlorpromazine
